# Supplementary figures and images for: A Bioinformatics Evaluation of the Role of Dual-Specificity Tyrosine-Regulated Kinases in Colorectal Cancer
Source: Cancers (Basel). 2022 Apr 18;14(8):2034. doi: 10.3390/cancers14082034 (PMC9025863; doi:10.3390/cancers14082034)

**Figure S1**

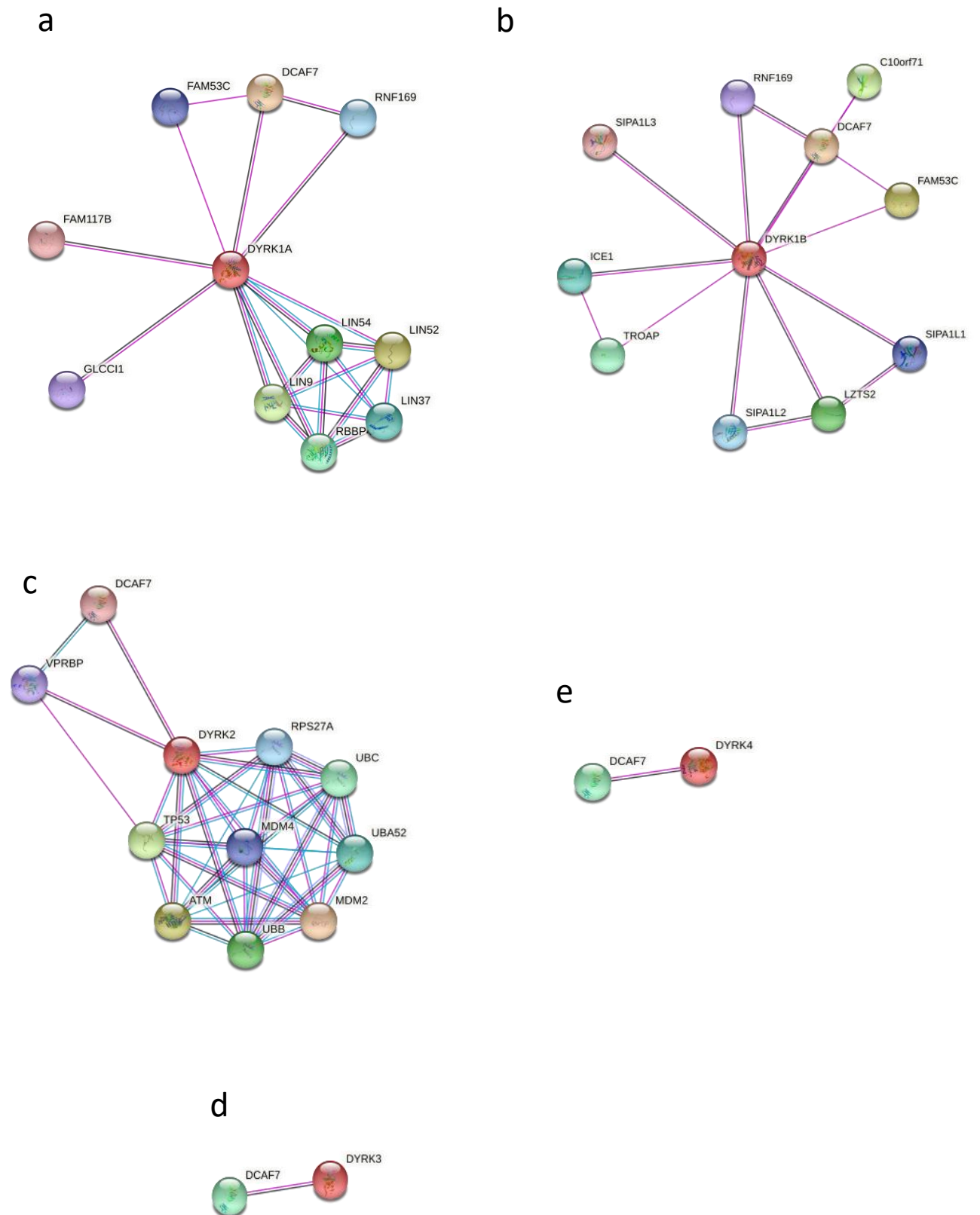

**Figure S1:** Functional protein association network predicted from STRING.

Supplement: Supplementary file 1 [file cancers-14-02034-s001.zip › cancers-1671932-supplementary.pdf]
